# Physiochemical and Microbial Analysis of Tibetan Yak Milk Yogurt in Comparison to Locally Available Yogurt

**DOI:** 10.3390/molecules28135242

**Published:** 2023-07-06

**Authors:** Zhipeng Huang, Arslan Habib, Xiaoming Ding, Hong Lv

**Affiliations:** 1 Collaborative Innovation Center for Genetics and Development, State Key Laboratory of Genetic Engineering, Department of Microbiology, School of Life Sciences, Fudan University, Shanghai 200438, China; 2 Laboratory of Molecular Immunology, State Key Laboratory of Genetic Engineering, School of Life Sciences, Fudan University, Shanghai 200438, China; 3 State Key Laboratory of Genetic Engineering, School of Life Sciences, Fudan University, Shanghai 200438, China; 4 Shanghai Engineering Research Center of Industrial Microorganisms, Fudan University, Shanghai 200438, China

**Keywords:** genetic stability, Tibetan yak yogurt, fermented milk, *Lactobacillus bulgaricus*, *Streptococcus thermophilus*

## Abstract

Yak yogurt, which is rich in microorganisms, is a naturally fermented dairy product prepared with ancient and modern techniques by Chinese herdsmen in the Qinghai-Tibet Plateau. The objective of this research was to assess the impact of *Lactobacillus bulgaricus* and *Streptococcus thermophilus* starter cultures on the quality and shelf life of yak yogurt, as well as the genetic stability across multiple generations, in comparison to commercially available plain yogurt and peach oat flavor yogurt. Following that, the samples were evenly divided into four treatment groups denoted as T1 (treatment 1), T2, T3, and T4, with each group employing a distinct source of yogurt formulation. T1 included *L. bulgaricus*, T2 comprised *S. thermophilus*, T3 consisted of plain yogurt, and T4 represented peach oat yogurt flavor. The findings indicate that T1 yogurt consistently presents a lower pH and higher acidity compared to the other three yogurt types throughout the entire generation process. Moreover, the fat content in all generations of the four yogurt types exceeds the national standard of 3.1 g/100 g, while the total solid content shows a tendency to stabilize across generations. The protein content varies significantly among each generation, with T1 and T4 yogurt indicating higher levels compared to the T2 and T3 yogurt groups. In terms of overall quality, T1 and T4 yogurt are superior to T2 and T3 yogurt, with T1 yogurt being the highest in quality among all groups. The findings revealed that the inclusion of *L. bulgaricus* led to enhanced flavor, texture, and genetic stability in yak yogurt. This study will serve as a valuable source of data, support, and methodology for the development and screening of compound starters to be utilized in milk fermentation in future research and applications.

## 1. Introduction

In recent decades, there has been a surge in the demand for better health, leading to an increase in the production of nutritious and functional foods. Among the healthy and functional foods, fermented dairy products are known for their numerous health benefits derived from the natural fermentation of lactic acid bacteria [1]. Yogurt serves as a vital source of essential macro- and micronutrients within the fermented diet, are rich in proteins, lipids, and minerals [2]. The fact that yogurt is cherished and widely consumed as a culinary staple around the world makes a key component of a healthy diet [3]. As science and technology advance, the production scale of yogurt has continued to expand, and fermentation techniques have become increasingly cultured and refined.

Yaks are predominantly distributed in high-altitude areas of the Qinghai-Tibet Plateau and alongside alpine regions in China, with a population of 3.9 million, constituting approximately 30% of the global yak population. The lactation period of yaks is characterized by a remarkable milk yield, ranging from 450 to 600 kg, showing the impressive productivity of these resilient animals [4]. Yaks play a vital role in the livelihood of Tibetan herdsmen, serving as a valuable source of livestock, meat, fur, and other livestock products. Their contribution to the local economy is significant, providing essential resources that sustain the livelihoods of the local people [5]. Yak milk, in comparison to commercially available milk, is abundant with protein, essential amino acids, lactose, fatty acids, lactoferrin, conjugated linoleic acid, and minerals. When used as the raw material, fresh yak milk gives rise to traditional fermented yak yogurt, a unique and flavorful dairy product with low alcohol content. This yogurt is produced using a complex microbial system that incorporates diverse lactic acid bacteria [6]. The research conducted in the pastoral area of Yushu Tibetan Autonomous Prefecture, Qinghai, China, has revealed the presence of various lactic acid bacteria, including *Lactobacillus delbrueckii*, *Bacillus*, *Lactococcus*, *Enterococcus*, and others in solidified yak yogurt [7]. Different types of lactic acid bacteria have been identified in Tibet’s traditional fermented such as *L. paracasei*, fermentation of *L. paracasei*, *Limosilactobacillus fermentum*, *Lactobacillus plantarum*, among others, with *Lactobacillus delbrueckii* being the dominant bacteria. These findings shed light on the complex microbial diversity and fermentation process of this traditional yak milk, highlighting the significant presence of *Lactobacillus delbrueckii* as a key contributor to its unique characteristics and flavors [8]. These strains of lactic acid bacteria not only possess various beneficial properties such as cholesterol-lowering, antioxidant effects, and the potential to promote healthy bowel movements, but they also contribute to the distinct flavor and high permissibility of yak yogurt. Indeed, the strains of lactic acid bacteria present in yak yogurt play a significant role in determining the quality and probiotic characteristics of yogurt [9].

Currently, due to resource and geographical limitations, the yak milk resources have not been rationally developed and utilized, leading to a relatively weak industrial production of yak yogurt at present. So far, the Chinese dairy product fermentation industry relied on imported yogurt fermentation agents with limited utilization of local fermentation agents. Therefore, it is imperative to focus on the research and development of localized, high-efficiency fermentation agents and the production of superior-quality fermented milk to benefit China’s dairy fermentation industry significantly [9]. The current study emphasizes meeting the needs of consumers by producing and processing high-quality yak yogurt while exploring the genetic stability of Tibetan yak yogurt. The objectives of this study were to compare two strains of Tibetan yak yogurt with two commercially available yogurts at different levels, analyzing powder yield, viable lactic acid bacteria count, and the pH of each yogurt type were measured.

## 2. Results and Discussion

### 2.1. Viable Counts during Yak Yogurt Generation

**Figure 1:**
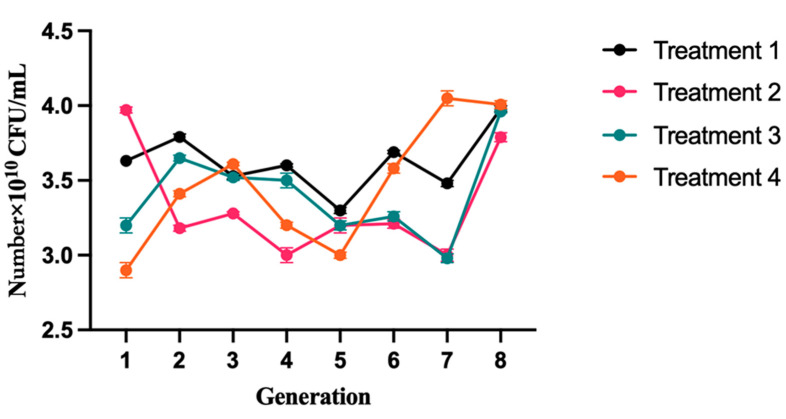
Changes in microbiology during the yak milk fermentation process. The curves in different colors represent distinct groups, depicting changes in viable cell count and their corresponding variations over time. Significantly different (*p* < 0.05) values are indicated among the groups.

The microbial content of yogurt from each treatment was analyzed, and the results were compared to those of the control group. The viable counts of lactic acid bacteria (LAB) in the yogurt were represented as the cumulative sum of LAB counts. Based on the data presented in Figure 1, it is evident that all four types of yogurts exhibit a significant abundance of viable lactic acid bacteria, ranging from 2.98 × 10^10^ to 4.05 × 10^10^ CFU/mL, surpassing the national standard of 1 × 10^6^ CFU/mL (National food safety standard, GB 19302-2010). This indicates that the yogurt is in a favorable state of fermentation during the passage culture process. The average viable count of lactic acid bacteria in each generation of T1 yogurt is 3.63 × 10^10^ CFU/mL, in T2 yogurt, is 3.33 × 10^10^ CFU/mL, in T3 yogurt is 3.41 × 10^10^ CFU/mL, and in T4 yogurt is 3.47 × 10^10^ CFU/mL. The viable bacterial counts in the T1 and T4 groups were significantly higher (*p* < 0.05) compared to the T2 and T3 groups during the different generation periods. One possible explanation could be that *Lactobacillus bulgaricus* (*L. bulgaricus*) is better suited for growth in yak milk, resulting in higher viable bacterial counts in the T1 group compared to the T2, T3, and T4 groups during the different generation periods.

Ranadheera et al. (2010) [10] found that the survival of probiotic microorganisms in food products is influenced by various properties of the food product, such as protein concentration, fat content, pH, as well as storage conditions. Compared to regular milk, yak milk has a distinctive composition. Higher quantities of total solids, fat, and protein are known to be present. These elements give bacteria, particularly LAB, a rich source of nutrients that encourages their development. Yak milk’s microbial composition may change according to unique environmental factors, such as cold temperatures and high altitudes. LAB are more suited than other bacteria to survive in these circumstances and are more tolerant of low temperatures. Natural fermentation techniques used in traditional milk processing encourage the formation of LAB. The majority of the microflora in yak milk is thought to be LAB [11,12]. The T4 group, which consists of commercially flavored yogurt, introduces additional factors that could potentially influence the survival of bacteria. These factors should be taken into consideration when interpreting the observed results. The data indicates that the average viable count of lactic acid bacteria in T1 yogurt is higher compared to the other groups, suggesting that the fermentation of T1 yogurt was the most successful among all the yogurt groups. As per Iravani et al. (2015), the primary factors that can limit the stability of lactic acid bacteria in fermented products are the low pH and titrable acidity levels of the products [13]. As a pivotal parameter for evaluating viability, the viable cell counts of lactic acid bacteria are considered significant in the production of fermented dairy products. Typically, it is recommended to maintain viable cell counts in yogurt at 10^7^ CFU/mL or higher [14]. The findings of the present study align with this standard, confirming similar results.

### 2.2. Fat Content Analysis in Yogurts Evaluated

**Figure 2:**
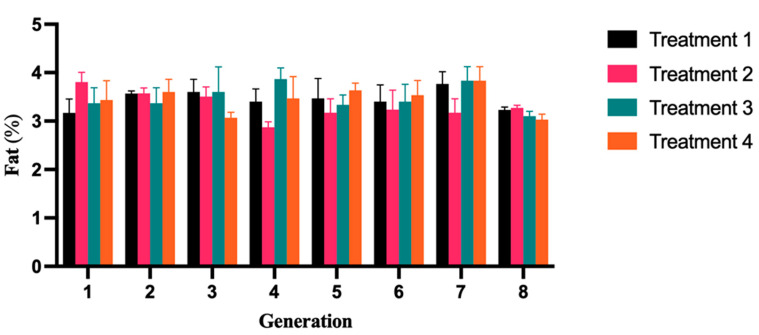
Changes in the fat content of fermented yak milk across various generation times. The graph represented the significant trend of fat variation in four groups of fermented milk samples across eight levels. A significant difference was observed among the groups. The error bars represent the standard deviation (SD).

Dairy products that are rich in short-chain fatty acids with a carbon chain length ranging from C4:0 to C10:0 are readily absorbed by the body [15]. In comparison to other treatment groups, the amount of short-chain fatty acids in yak yogurt T2 was considerably significant (*p* < 0.05), particularly butyric acid and hexanoic acid. Yak yogurt had considerably less medium-chain fatty acid than other treatment groups (*p* < 0.05). In the yogurt samples, palmitic acid, stearic acid, and oleic acid were found to be the primary long-chain fatty acids. Based on Figure 2, the average fat content of the four types of yogurts in each generation is as follows: T2 (3.32%) < T4 (3.42%) < T1 (3.43%) < T3 (3.45%). These values are higher than the national standard by 3.1% (g/100 g). The average fat content of T2 yogurt is the lowest among the four types of yogurts, and the fifth generation of fermented T2 yogurt indicates the lowest fat content of 2.87%. This value is not only the lowest among all recorded fat values but also falls below the national standard (Table 1). This finding suggests that the fat content of T2 yogurt, in terms of its physical and chemical properties, does not confer an advantage compared to the other four types of fermented yogurts. Notably, there is a noticeable disparity, particularly among the other three groups. Güler and Gürsoy-Balcı (2011) also noted that the addition of varying proportions of starter cultures, CH-1 (*Streptococcus thermophilus*) and YF-3331 (*Lactobacillus delbrueckii* ssp. bulgaricus), did not impact the content of medium-chain fatty acids (C12–C15:1) in goat yogurt [16]. As per the guidelines provided by the USDA and FDA, yogurt is classified as nonfat, low-fat, or regular based on its fat content, with less than 0.5%, 0.5–2.0%, and at least 3.25% of fat, respectively [17]. The results indicate that our yogurt falls into the category of regular yogurt.

### 2.3. Characterization of pH and Acidity in Yogurts Evaluated

**Figure 3:**
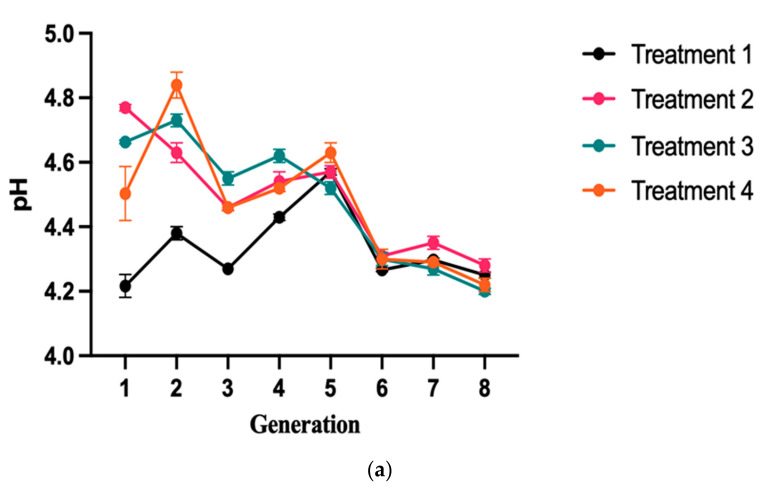

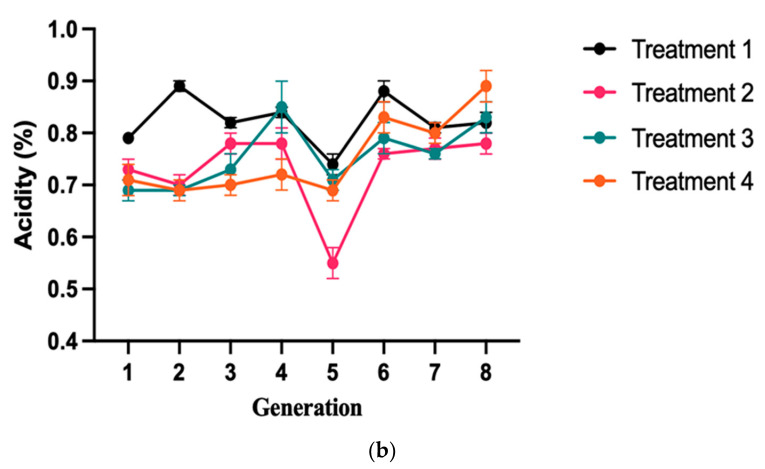
Variations in pH and acidity of fermented yak milk across different generations. (**a**) A significant trend of pH variation in four groups of fermented yak milk samples across different generations was observed. The pH changes in different groups were indicated by broken lines of varying colors. Among groups T1, T2, T3, and T4, a significant difference was observed, with the lowest pH value of 4.22 (*p* < 0.05) being recorded in T1. The pH values are presented as mean (±SD). (**b**) The trends in the acidity of four groups of fermented yak milk samples across different generations. The broken lines of varying colors indicated the changes in the acidity of different groups across different levels. There were significant differences observed between the groups, with group T1 showing significant differences from the other three groups across different levels. The acidity values are presented as mean ± SD.

The pH value and TA acidity play a significant role in the viability of lactic acid bacteria during fermentation and storage, as well as the quality of fermented dairy products. The main function of a fermented milk starter is to initiate and facilitate the production of lactic acid through the metabolic activity of lactic acid bacteria during the fermentation process [18]. The pH quality parameters for T1, T2, T3, and T4 were recorded concurrently. Figure 3A illustrates the pH variations among different treatment groups with varying generations (*p* < 0.05). Based on the statistical analysis, the pH values of T1, T2, T3, and T4 were found to be significantly different. The pH values of T1 were found to be significantly lower than those of other treatment groups (*p* < 0.05). As per Jovanović M et al. (2021) [19], lactic strains possess the ability to ferment lactose into lactic acid, which leads to an increase in acidity and a decrease in the pH of yogurt. The production of lactic acid ultimately results in a reduction in pH. During the first generation of yogurt in T1, the pH value was recorded as 4.22, which is lower than the pH values of T2 (4.77), T3 (4.66), and T4 (4.50), respectively (Table 2). The average pH value for eight generations, from the first to the eighth generation, was 4.34 for T1, 4.47 for T4, 4.48 for T3, and 4.49 for T2. The average pH value of T1 yogurt is lower than that of the other treatment groups, indicating that lactic acid bacteria quickly become the dominant group during the fermentation process of T1 yogurt, resulting in better fermentation quality compared to the other treatment groups of yogurts. Undoubtedly, pH and acidity control are significant parameters in yogurt processing, as they play a significant role in curd coagulation, ripening, and shelf life of the final product [20]. According to the present study, the pH value of the T1 group decreased as a result of the addition of lactic acid bacteria, consistent with the findings of Akgun et al. (2018) [21]. In their study, Akgun et al. observed that the pH values of the milk during processing, from the time of inoculation with bacterial cultures to the time of yogurt manufacturing, decreased from 6.70 to 4.34. It has been demonstrated that the pH value typically decreases to a range of 4–5 during the yogurt fermentation process, which is attributed to the production of lactic acid that lowers the pH. At the isoelectric pH (pH 4.6) approach, the casein micelles lose their steric stability, resulting in their flocculation, precipitation, and subsequent formation of a coagulum, as noted by Loveday et al. in 2013 [22]. The decrease in pH during yogurt fermentation is attributed to the activity of microorganisms that utilize residual carbohydrates and produce lactic acid, small amounts of CO_2_, and formic acid, as reported by Vital et al. in 2015 [23].

The acidity analysis was conducted by calculating the mean values of different treatment groups across different generations simultaneously. The results were analyzed using one-way ANOVA, and the means were compared accordingly. Figure 3B shows that the average acidity values of T1, T2, T3, and T4 yogurts during the passage process were 0.82%, 0.75%, 0.76%, and 0.75%, respectively. The average acidity value of T1 yogurt was observed to be higher than that of the other yogurt groups (Table 3). According to Servili et al. (2011), the elevation in acidity levels can be attributed to the growth and activity of lactic acid bacteria [24]. In the control condition, the commercially available plain yogurt, which did not undergo any special treatment, showed nonsignificant results over time. However, it was also observed that the peach oat flavor yogurt demonstrated significant acidic values (Table 3). Vinderola et al. (2019) reported that the co-fermentation of *L. bulgaricus* and the starter could reduce the postacidification of yogurt and improve the stability of products during storage [25]. Typically, there exists an inverse relationship between pH and acidity, with lower pH values indicating higher acidity levels. Throughout the yogurt fermentation process, a general trend of decreasing pH values accompanied by increasing acidity levels was observed [26]. As suggested by Wang et al. (2021), this phenomenon may be attributed to the continuous production of lactic acid and other organic acids resulting from the consumption of lactose by lactic acid bacteria and starter cultures [27].

### 2.4. Characterization of Physiochemical Properties in Yogurts Evaluated

**Figure 4:**
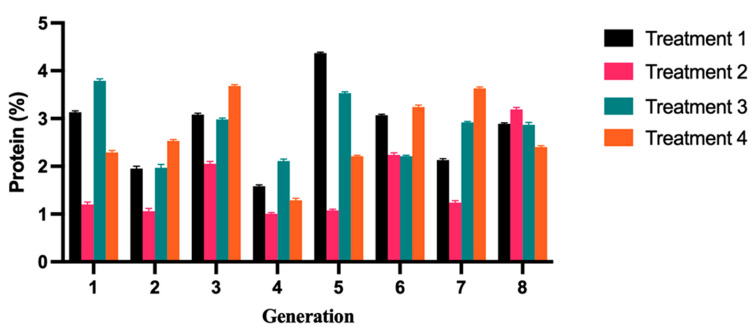
Variations in protein content of fermented yak milk. The protein content level was observed in four different treatment groups, with the average protein content of T2 yogurt being the lowest and significantly lower compared to the other three types of yogurts (*p* < 0.05). The error bars represent the mean SD.

Indeed, Figure 4 reveals notable variations in protein content as the different types of yogurts progress through the fermentation process. This underscores how protein content can effectively reflect the nutritional value of yogurt, serving as an important parameter for evaluating its quality [28]. The average protein content for each generation of T1 yogurt was 2.78%; for T2 yogurt, it was 1.63%; for T3 yogurt, it was 2.80%; for T4 yogurt, it was 2.66%. The average protein content of T2 yogurt is the lowest, and it is significantly lower compared to the other three types of yogurts (Table 4). Proteins can act as emulsifiers, enveloping oil droplets and providing steric stabilization to prevent their aggregation [29]. Hence, a higher protein content could result in increased availability of emulsifiers to stabilize lipid droplets in the system [30]. It should be noted that higher protein content was associated with more sedimentation. The average protein content of T1, T3, and T4 yogurts in each generation was 2.3% higher than the national standard protein content. Notably, the protein content of T2 and T3 yogurts was higher compared to that of T2 and T4 yogurt groups. While it is important to obtain essential amino acids from food, it is crucial to consume them in the correct proportions to ensure proper nutrition and support optimal bodily functions [31]. Previous studies have shown that the amino acid profiles and peptide sequences are vital factors influencing certain biological activities [32].

**Figure 5:**
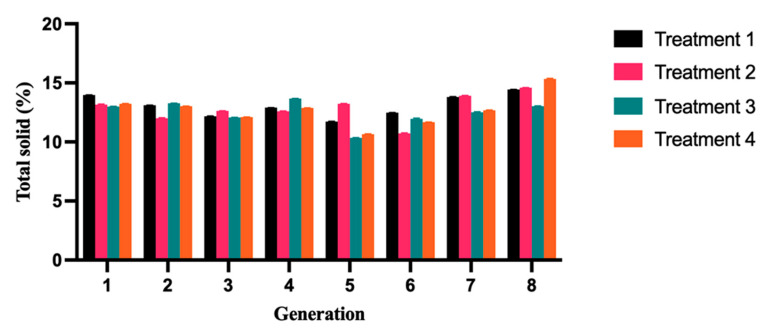
Changes in solid content during the yak milk fermentation process. A significant difference was found between group T1 and groups T2, T3, and T4. The T1 yogurt showed the highest average total solid content of 13.08%, indicating a desirable solidification state during the fermentation process. Mean (±SD) values of solid contents.

Based on the data presented in Figure 5, it is evident that the total solid content of four distinct types of yogurts tends to stabilize across generations during the passage process, with minor differences observed between different generations. The average total solid content of T1 yogurt in each generation is 13.08%, T2 yogurt is 12.85%, T3 yogurt is 12.48%, and T4 yogurt is 12.70% (Table 5). The T1 yogurt showed the highest average total solid content, indicating a desirable solidification state during the fermentation process. Total solids play a critical role in determining the quality and stability of yogurt. As reported by Rodriguez et al. (2017), slight increases in total solids were observed in all samples during a storage period of up to 60 days. This increase was attributed to partial losses in free water that occurred during storage [33]. Yak milk, due to its higher total solid content, has been shown to enhance the gel strength of yogurt and reduce pore size, resulting in better water retention and reduced syneresis, as reported by Moreno-Montoro et al. in 2018 [34].

### 2.5. Characterization of Powder Yield in Yogurts Evaluated

**Figure 6:**
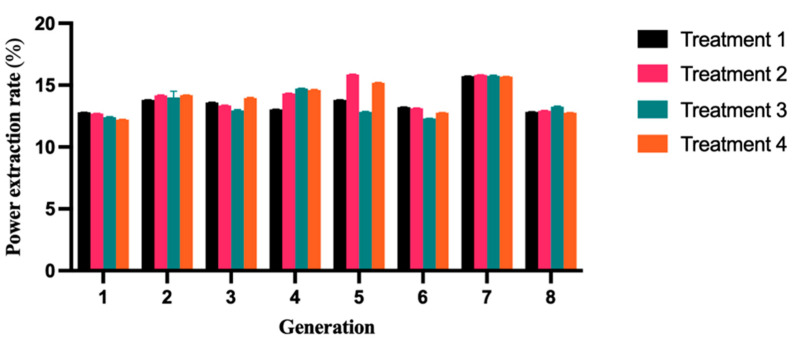
Different yogurt types are characterized based on their powder yield. The stability of the fermented milk is significantly improved during storage by T1 yogurt, leading to an overall improvement in the quality of the product. The error bars represent the mean SD.

Based on Figure 6, it is evident that the four different types of yogurts tend to stabilize at similar levels across generations during the passage process, with minimal variation observed between different generations, with values ranging from 12.77% to 15.85% (Table 6). During storage, T1 yogurt greatly enhances the stability of the fermented milk, thereby elevating the overall quality of the product. For an ideal yak yogurt, it should possess a desirable viscosity, consistent texture, and unwavering stability. Thus, this implies that the process of yogurt fermentation is notably stable, exhibiting minimal deviation and exemplary passage stability.

### 2.6. Sensory Evaluation of Yak Yogurt

**Figure 7:**
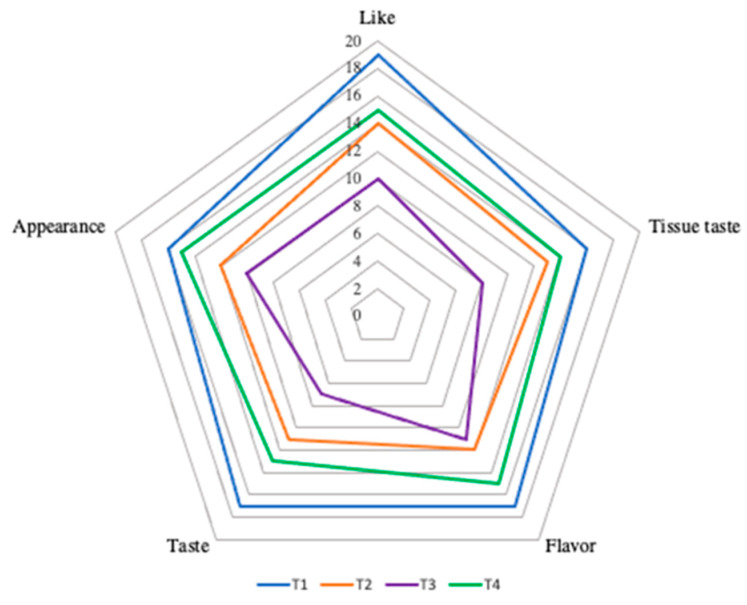
The direct view of sensory evaluation of fermented yak milk is depicted with different color broken lines, representing the scores from various groups in five different aspects: appearance, taste, flavor, tissue state, and overall liking.

Sensory evaluation of food relies on the human body’s five senses to assess various indicators of the product. The sensory evaluation method is considered practical and reliable, as it plays a crucial and intuitive role in food evaluation. Presently, no technology can fully replicate the sensory capabilities of human sense organs in the evaluation of food products. The four yogurts exhibited differences in terms of appearance, taste, flavor, texture, and overall preference. The scores for fermented milk appearance were as follows: T1 > T2 > T3 > T4, with the T1 and T2 groups showing the highest and most similar values. In terms of mouthfeel, the smoothness of fermented milk ranked as follows: T1 > T2 > T3 > T4, with the T1 group exhibiting the smoothest mouthfeel (Figure 7). In terms of flavor, group T1 was noted to have a pleasant taste without any unpleasant odor. The flavor scores of the yak yogurt groups T2 and T4 were closely matched, and group T3 had the lowest flavor score. Consumers can assess the quality of the product by evaluating its sensory characteristics, including appearance, flavor, texture, and odor. After being stored at 4 °C for 1 day, it was determined that the products from several groups did not show any quality defects, such as unpleasant odor or texture.

The fermented milk in group T1 exhibited a smooth taste, delicate texture, and pleasant flavor, which were well-received by people. The favorable sensory quality observed in groups T1 and T2 indicated that the *L. bulgaricus* and *S. thermophilus* strains determined for this analysis benefited by enhancing the flavor and quality of the fermented milk. The interaction mechanism of these strains in the yogurt relied on the utilization of metabolites and growth-promoting factors that were produced through their metabolism. The sensory characteristics of the fermented milk varied as a result of the unique strains and their proportions chosen by different starter cultures [35]. Research conducted by Jiale in 2021 revealed that varying the blending ratio of *L. bulgaricus* IMAU 20,312 and *S. thermophilus* IMAU80809 had a significant influence on the fermentation characteristics and sensory quality of fermented milk, as evidenced in the findings of the study [36]. Skriver et al. (2003) [37] suggested that combining various strains of lactic acid bacteria could result in diverse fermentation performances, as proposed in their research. According to Urshev et al. 2006 [38], the fermentation process of yogurt is closely associated with the combination of different strains of *L. bulgaricus* and *S. thermophilus* in the starter culture, resulting in desirable characteristics such as rapid fermentation and distinctive flavor profiles [38]. The fermentation of Tibetan yak milk yogurt can result in the production of several secondary metabolites. These metabolites help give the yogurt its distinct flavor, taste, and health advantages. The main metabolite produced during yogurt fermentation is lactic acid. Acetaldehyde is a result of lactic acid bacteria fermenting lactose. Moreover, diacetyl is a chemical with a buttery flavor that can be formed during fermentation by several lactic acid bacteria *S. thermophilus*. Another substance that adds to the buttery flavor of yogurt is acetoin. Some types of environmental yeast may turn carbohydrates into ethanol during the early stages of fermentation. During fermentation, certain lactic acid bacteria create bacteriocins, which are antibacterial peptides. These peptide antibacterial characteristics aid in preventing the development of harmful bacteria in yogurt, aiding in its preservation and safety [39,40].

## 3. Materials and Methods

### 3.1. Tibetan Yak Yogurt Preparation

Tibetan yak milk was procured directly from a local herdsman family residing in the Qinghai Tobet Plateau in Yushu, Qinghai, China. For this experiment, the raw material utilized was Tibetan pasteurized yak milk which was used to prepare the yogurt. Following that, the samples were evenly divided into four treatment groups denoted as T1 (treatment 1), T2, T3, and T4, with each group employing a distinct source of yogurt formulation. T1 included *Lactobacillus bulgaricus*, T2 comprised *Streptococcus thermophilus*, T3 consisted of plain yogurt, and T4 represented peach oat flavor yogurt. Commercially available plain yogurt and peach oat flavored yogurt were used as comparison samples to evaluate the potential of the designed strains and the quality of Tibetan yak yogurt.

**Figure 8:**
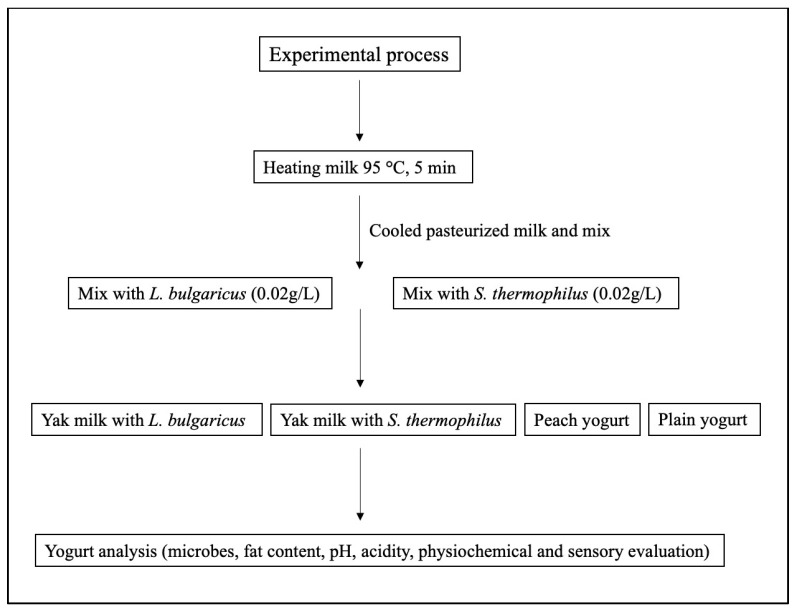
Experimental procedure of different yogurt preparation and its analysis.

The conventional yak yogurt samples were used as a reference standard in comparison to other commercially available yogurts used in the present investigation. The yak milk was heated to a temperature of 95 °C for 5 min using a water bath. The cooled pasteurized milk was mixed with *L. bulgaricus* (0.02 g/L) and *S. thermophilus* (0.02 g/L) and subsequently incubated at a temperature of 43 °C for 12 h until the pH level reached the range of 4.6 to 4.5, ensuring optimal conditions for the desired outcome. The yogurt samples were made according to the method described in [41,42] with some modifications. Four distinct varieties of yogurt were carefully prepared: yak yogurt with *L. bulgaricus*, yak yogurt with *S. thermophilus*, and two commercially available plain yogurt and peach oat flavored yogurt were directly obtained from the market. Ultimately, the products were stored under refrigeration at a temperature of 4 ± 1 °C to facilitate further analysis. Following that, they were kept at 10 °C for 21 days while reading was obtained based on different parameters (Figure 8). On the first day of storage, fatty acid, flavor, storage, and sensory analyses were conducted on the samples. Yogurts were prepared in triplicate throughout three independent sessions.

The enumeration of lactic acid bacteria was conducted using the method outlined by the International Dairy Federation as described in the research protocol [43]. The pure strain was streak cultured on De Man, Rogosa, and Sharp (MRS) medium for 48 h at 30 °C. Subsequently, colony size and shape were thoroughly observed and tested through various methods, including the microscopic examination (BX43, Olympus, Tokyo, Japan), Gram-staining, catalase test, reduction test, litmus milk test, growth temperature test (10 °C, 15 °C, 45 °C, and 60 °C for 30 min) and pH gradient test, all conducted with high precautions. The enumeration of *Strep. thermophilus* colonies were conducted using an M17 medium with a pH of 7.0. The colony-forming units (CFU) per milliliter (mL) of yogurt were used to express the microbiological count data.

### 3.2. Fatty Acid Analysis

The fatty acid analysis of the yogurt samples was examined by GC-MS (Agilent Technologies Inc. Santa Clara, CA) following the method outlined by Ajmal et al. (2019) [44]. In brief, the yogurt samples were subjected to total lipid extraction using a chloroform: methanol (2:1) mixture. Subsequently, 30 μL of the extracted lipid was transferred to a 10-mL centrifuge tube, and 2 mL of hexane and benzene mixed reagent (1:1) was added and gently shaken to facilitate dissolution. Next, 2 mL of a solution containing 0.5 mol/L potassium hydroxide in methanol was added to the mixture, and the contents were shaken. The tube was allowed to stand at room temperature for 30 min, following which distilled water was added to cause the methanol solution in the organic phase to separate and rise to the top of the tube. Lastly, the supernatant was carefully extracted from the top layer after allowing the mixture to stand for 10 min in preparation for further analysis. The extracted samples were subjected to analysis using GC-MS (Agilent Technologies Inc., Santa Clara, CA, USA) with a DB5 capillary column (J&W Scientific, Agilent Technologies Inc., Santa Clara, CA, USA). The identification and quantification of individual fatty acids in the samples were accomplished by comparing their retention times and peak areas with those of corresponding standards.

### 3.3. Determination of pH and Acidity

The digital pH meter (Mettler Toledo, Columbus, OH, USA) was utilized to assess the pH of the yogurt samples, while the acidity of the yogurts was assessed through titration using 0.1 mol/L NaOH with phenolphthalein as an indicator, following the methodology determined as described [45].

### 3.4. Physiochemical Determinations

Standardized instrumental methods were employed to determine the physiochemical parameters of yogurt samples during cold storage. The total solids of the samples were determined at different storage times as described [45]. The protein content was estimated by determining the total nitrogen level using the Kjeldhal method, with a conversion factor of N × 6.38 providing a precise measurement of the protein content [46]. All four varieties of yogurt were dried using the freeze-drying technique after making the required changes. A laboratory freeze dryer (ALPHA 1-2 LD Plus, Osterode am Harz, Germany) was used to achieve this. After drying, the powders were collected and stored in aluminum pouches at room temperature. The final dried powder was evaluated for further structural analysis and sustainability in different generations.

### 3.5. Sensory Evaluation

Twelve students were chosen to form a sensory evaluation group, and they received training that was relevant to the field of sensory evaluation. After the completion of fermentation and postmaturation at 4 °C for 1 day, the fermented milk was subjected to sensory evaluation, with each sample rated on a scale of 10 points. The fermented milk was presented for sensory evaluation after fermentation completion and postmaturation at 4 °C for 1 day, with each item receiving 20 points. A trained panel of four judges, consisting of two men and two women ranging in age from 25–35 years, assessed the sensory qualities of yogurts. The judges received two training sessions from professionals to evaluate the sensory qualities of yogurt. Following the outlined technique, each assessor received papers with a 20-point scale for each quality (flavor, mouthfeel, appearance, liking, and taste). Table 7 displays the sensory evaluation criteria used for assessing the fermented milk samples [47].

### 3.6. Statistical Analysis

The data set was analyzed through one-way ANOVA employing the Statistical Package for Social Science (SPSS 18.0, SPSS, Inc., Chicago, IL, USA). When the overall treatment effect was observed to be significant, a Tukey’s test was used to conduct a pairwise comparison among the means of the treatments. The least squares mean and standard error of the means were reported for each treatment, and significant differences among the means were determined at a significance level of *p* < 0.05.

## 4. Conclusions

In this study, an evaluation was conducted on the performance of two strains of lactic acid bacteria, namely *L. bulgaricus* and *S. thermophilus*, for passage stable fermentation culture, using commercially available plain yogurt and peach oat flavor yogurt as the fermentation substrates. Throughout eight consecutive passages of culture, the physical and chemical properties of the four types of yogurts were assessed using various methods, including dilution, plate line separation and culture, basic titration, Coomassie brilliant blue staining spectrophotometry, and other analytical techniques. The test results showed that the pH value of T1 yogurt was consistently lower than the other three types of yogurts throughout the fermentation process, and the acidity was higher in comparison to the other three types of yogurts. The number of viable lactic acid bacteria in each generation of the four types of yogurts during the fermentation process exceeds the national standard by 1 × 10^6^ CFU/mL. Moreover, the average fat content of each generation surpasses the national standard by 3.1%. Additionally, the average number of live lactic acid bacteria in T1 yogurt is higher compared to the other groups of yogurts, indicating that the fermentation quality of T1 yogurt is superior to the other three types of yogurts. The total solid content and powder yield of the four types of yogurts showed a tendency to stabilize during the fermentation process, suggesting good stability in the passage process. Additionally, the inclusion of LAB in fermented yak milk yogurt products has a significant impact on factors such as acidification, sensory attributes, viable LAB counts, and protein concentration. This study establishes a theoretical foundation and serves as a valuable data reference for future investigations aimed at developing lactic acid bacteria starter cultures using specific lactic acid bacteria strains.

## Figures and Tables

**Table 1:** The fat content in different types of yogurts in different generations.

Generation	T1	T2	T3	T4	SEM ^1^	*p*-Value
1	3.17 ± 0.02 ^b^	3.80 ± 0.01 ^a^	3.37 ± 0.02 ^ab^	3.43 ± 0.01 ^ab^	0.10	0.172
2	3.57 ± 0.03	3.57 ± 0.01	3.37 ± 0.01	3.6 ± 0.03	0.06	0.568
3	3.6 ± 0.01	3.5 ± 0.02	3.6 ± 0.03	3.07 ± 0.01	0.10	0.196
4	3.40 ± 0.02 ^b^	2.87 ± 0.01 ^c^	3.87 ± 0.01 ^a^	3.77 ± 0.01 ^ab^	0.13	0.002
5	3.47 ± 0.01	3.17 ± 0.01	3.33 ± 0.02	3.63 ± 0.03	0.08	0.263
6	3.23 ± 0.03	3.27 ± 0.03	3.1 ± 0.01	3.03 ± 0.01	0.05	0.426
7	3.77 ± 0.01 ^a^	3.17 ± 0.01 ^b^	3.83 ± 0.03 ^a^	3.83 ± 0.02 ^a^	0.11	0.051
8	3.23 ± 0.02	3.27 ± 0.02	3.10 ± 0.01	3.03 ± 0.01	0.05	0.426

^a,b,c^ Means within a row with different superscripts differ (*p* < 0.05). ^1^ SEM, standard error of the means.

**Table 2:** The pH in different types of yogurts in different generations.

Generation	T1	T2	T3	T4	SEM ^1^	*p*-Value
1	4.22 ± 0.01 ^d^	4.77 ± 0.02 ^a^	4.66 ± 0.01 ^b^	4.50 ± 0.03 ^c^	0.06	<0.001
2	4.38 ± 0.02 ^c^	4.63 ± 0.01 ^b^	4.73 ± 0.03 ^ab^	4.84 ± 0.02 ^a^	0.06	0.003
3	4.27 ± 0.02 ^c^	4.46 ± 0.02 ^b^	4.55 ± 0.01 ^a^	4.46 ± 0.02 ^b^	0.03	<0.001
4	4.43 ± 0.01 ^c^	4.54 ± 0.04 ^b^	4.62 ± 0.02 ^a^	4.52 ± 0.03 ^b^	0.02	<0.001
5	4.57 ± 0.03 ^ab^	4.57 ± 0.01 ^ab^	4.52 ± 0.01 ^b^	4.63 ± 0.02 ^a^	0.02	0.125
6	4.27 ± 0.01	4.31 ± 0.01	4.30 ± 0.02	4.30 ± 0.02	0.01	0.235
7	4.30 ± 0.02 ^b^	4.35 ± 0.03 ^a^	4.27 ± 0.01 ^b^	4.29 ± 0.01 ^b^	0.01	0.004
8	4.25 ± 0.02	4.28 ± 0.01 ^a^	4.20 ± 0.03 ^b^	4.22 ± 0.01	0.01	0.065

^a,b,c^ Means within a row with different superscripts differ (*p* < 0.05). ^1^ SEM, standard error of the means.

**Table 3:** The acidity in different types of yogurts in different generations.

Generation	T1	T2	T3	T4	SEM ^1^	*p*-Value
1	0.79 ± 0.02 ^a^	0.73 ± 0.01 ^ab^	0.69 ± 0.01 ^ab^	0.71 ± 0.03 ^b^	0.02	0.099
2	0.89 ± 0.02 ^a^	0.70 ± 0.02 ^b^	0.69 ± 0.04 ^b^	0.69 ± 0.03 ^b^	0.03	0.01
3	0.82 ± 0.01 ^a^	0.78 ± 0.01 ^a^	0.73 ± 0.01 ^b^	0.70 ± 0.01 ^b^	0.02	0.004
4	0.84 ± 0.01 ^a^	0.78 ± 0.02 ^b^	0.85 ± 0.01 ^a^	0.72 ± 0.02 ^c^	0.02	<0.001
5	0.74 ± 0.03 ^a^	0.55 ± 0.01 ^b^	0.71 ± 0.03 ^a^	0.69 ± 0.02 ^a^	0.03	0.014
6	0.88 ± 0.01	0.76 ± 0.03	0.79 ± 0.02	0.83 ± 0.01	0.02	0.222
7	0.81 ± 0.02	0.77 ± 0.02	0.76 ± 0.01	0.80 ± 0.03	0.02	0.883
8	0.82 ± 0.01	0.78 ± 0.01	0.83 ± 0.02	0.89 ± 0.02	0.15	0.948

^a,b^ Means within a row with different superscripts differ (*p* < 0.05). ^1^ SEM, standard error of the means.

**Table 4:** The protein content in different types of yogurts in different generations.

Generation	T1	T2	T3	T4	SEM ^1^	*p*-Value
1	3.13 ± 0.01 ^b^	1.20 ± 0.01 ^d^	3.79 ± 0.03 ^a^	2.29 ± 0.02 ^b^	0.30	<0.001
2	1.95 ± 0.02 ^b^	1.06 ± 0.02 ^c^	1.97 ± 0.02 ^b^	2.53 ± 0.01 ^a^	0.16	<0.001
3	3.08 ± 0.01 ^b^	2.05 ± 0.03 ^c^	2.98 ± 0.02 ^b^	3.68 ± 0.03 ^a^	0.18	<0.001
4	1.58 ± 0.02 ^b^	1.01 ± 0.03 ^d^	2.11 ± 0.01 ^a^	1.29 ± 0.01 ^c^	0.12	<0.001
5	4.37 ± 0.03 ^a^	1.08 ± 0.01 ^d^	3.53 ± 0.03 ^b^	2.21 ± 0.02 ^c^	0.38	<0.001
6	3.07 ± 0.01 ^b^	2.24 ± 0.01 ^c^	2.21 ± 0.02 ^c^	3.24 ± 0.02 ^a^	0.14	<0.001
7	2.13 ± 0.02 ^c^	1.24 ± 0.04 ^d^	2.92 ± 0.01 ^b^	3.63 ± 0.01 ^a^	0.27	<0.001
8	2.89 ± 0.01 ^b^	3.19 ± 0.01 ^a^	2.87 ± 0.01 ^b^	2.43 ± 0.01 ^c^	0.08	<0.001

^a,b,c^ Means within a row with different superscripts differ (*p* < 0.05). ^1^ SEM, standard error of the means.

**Table 5:** The total solid content in different types of yogurts in different generations.

Generation	T1	T2	T3	T4	SEM ^1^	*p*-Value
1	13.97 ± 0.02	13.15 ± 0.01	12.99 ± 0.01	13.23 ± 0.02	0.21	0.404
2	13.10 ± 0.02	12.00 ± 0.01	13.27 ± 0.03	13.02 ± 0.01	0.22	0.166
3	12.18 ± 0.01	12.62 ± 0.03	12.07 ± 0.01	12.11 ± 0.05	0.21	0.817
4	12.90 ± 0.03	12.59 ± 0.01	13.67 ± 0.02	12.87 ± 0.01	0.23	0.417
5	11.73 ± 0.01 ^ab^	13.23 ± 0.02 ^a^	10.34 ± 0.01 ^b^	10.66 ± 0.03 ^b^	0.45	0.060
6	12.47 ± 0.01 ^a^	10.72 ± 0.02 ^b^	11.96 ± 0.02 ^ab^	11.67 ± 0.01 ^ab^	0.25	0.063
7	13.81 ± 0.02 ^a^	13.91 ± 0.01 ^b^	12.50 ± 0.02 ^b^	12.68 ± 0.01 ^b^	0.24	0.033
8	14.44 ± 0.01	14.59 ± 0.02	13.03 ± 0.01	15.34 ± 0.02	0.45	0.366

^a,b^ Means within a row with different superscripts differ (*p* < 0.05). ^1^ SEM, standard error of the means.

**Table 6:** The PER in different types of yogurts in different generations.

Generation	T1	T2	T3	T4	SEM ^1^	*p*-Value
1	12.80 ± 0.01	12.70 ± 0.01	12.40 ± 0.02	12.20 ± 0.03	0.20	0.367
2	13.81 ± 0.02 ^a^	14.18 ± 0.01 ^b^	14 ± 0.03 ^ab^	14.19 ± 0.01 ^b^	0.06	0.059
3	13.59 ± 0.02	13.36 ± 0.03	12.96 ± 0.01	13.95 ± 0.02	0.22	0.515
4	13.04 ± 0.02	14.33 ± 0.02	14.73 ± 0.02	14.61 ± 0.01	0.31	0.181
5	13.80 ± 0.02 ^b^	15.85 ± 0.01 ^a^	12.84 ± 0.02 ^b^	15.19 ± 0.02 ^a^	0.40	0.003
6	13.22 ± 0.03	13.13 ± 0.02	12.31 ± 0.01	12.77 ± 0.02	0.32	0.797
7	15.73 ± 0.01	15.82 ± 0.02	15.75 ± 0.03	15.69 ± 0.01	0.04	0.756
8	12.84 ± 0.01	12.93 ± 0.02	13.25 ± 0.02	12.77 ± 0.01	0.18	0.843

^a,b^ Means within a row with different superscripts differ (*p* < 0.05). ^1^ SEM, standard error of the means. PER, powder extraction rate.

**Table 7:** Fermented milk samples scoring criteria for sensory evaluation.

Sensory Features	15–20 Points	10–15 Points	0–10 Points
Like	Very like	Ordinary	No
Tissue taste	Fine and clot size if uniform	Fine tissue, uneven clot size	Rough tissue
Flavor	Good, no lousy smell	Good, no bad smell	Lousy smell
Taste	Smooth	Slightly smooth	Less smooth
Appearance	Smooth surface, no whey precipitation	Small amount of whey precipitated	Surface not smooth, large precipitation

## Data Availability

Not applicable.

## Sample Availability

Not available.
